# Editorial for the Special Issue on Silicon Photonics Bloom

**DOI:** 10.3390/mi11070670

**Published:** 2020-07-10

**Authors:** Qiancheng Zhao, Ozdal Boyraz

**Affiliations:** 1 Department of Electrical and Computer Engineering, University of California, Santa Barbara, Santa Barbara, CA 93106, USA; 2 Department of Electrical Engineering and Computer Science, University of California, Irvine, Irvine, CA 92697, USA

Silicon (Si) photonics debuted in the mid-1980s through the pioneering work done by Soref et al. While early work mainly focused on waveguides, switches, and modulators, significant momentum surged around the mid-2000s when great breakthroughs were achieved in GHz Si modulators, Raman Si lasers, and germanium (Ge)-on-Si epitaxial integration. Today, 30 years later, silicon photonics has experienced tremendous growth and become the backbone of integrated photonics, evolving from single passive components to hybrid functionalized architectures. Its scope is far beyond the traditional group IV elements, extending to compounds like silicon nitride, silicon oxynitride, and silicon carbon, in addition to heterogeneous integrations with III-V/II-VI elements, chalcogenide, graphene, crystals, polymers, etc. The popularity of Si photonics is partially attributed to its compatibility with the mature complementary metal–oxide–semiconductor (CMOS) technology that allows for low-cost and large-scale manufacturing. With the recent injection of government and private funding, more and more foundries, equipped with well-established and market-proven product development kits, will spring up, promoting a bloom in Si photonics in the new era. 

This Special Issue of *Micromachines*, entitled “Silicon Photonics Bloom”, has 10 research papers and 2 review articles, covering the scale from material preparation [1,2], to single device design [3,4,5,6,7], to photonic integration [8,9,10,11], to system architecture [12]. The demonstrated devices and components include source generation [1,5,6,11], modulators [7], switches [4,8], gratings [3,10], and couplers [9,12] and are applied to applications such as dispersion control [3], photonic memory [4], optic communication [8,10], polarization management [9], and photonic computing [12]. The spectrum of the contributed research spans a wide range, from visible [1,2,6], to telecom wavelength [3,4,7,8,9,10], to mid-IR [11], to terahertz frequencies [5].

Revolutionary technology usually starts from fundamental breakthroughs, especially in materials, in which new properties prompt unprecedented discoveries and innovations. Studies on the material properties are the cornerstones of silicon photonics, not only because new materials enable novel functionalities, but also because the accuracy of the material properties directly impacts the design of the photonic devices. Song et al. [1] studied SiC_x_O_y_ material, particularly on the effect of nitrogen doping on the photoluminescence of the amorphous SiC_x_O_y_ films. Nitrogen doping creates defect centers in the SiC_x_O_y_ bandgap. By varying the doping concentration, the defect center energy level could be adjusted, yielding photoluminescence from red to orange, as well as blue photoluminescence. Similar to the SiC_x_O_y_, the luminescent properties of the SiN_x_O_y_ film are also studied in this Special Issue. In the review article [2] by Shi et al., the luminescence properties and fabrication methods of the SiN_x_O_y_ films are summarized, and their applications as barrier materials in non-volatile semiconducting memory, optical devices, and anti-scratch coating are enumerated with abundant state-of-the-art examples. The review has an in-depth elaboration of the preparation of the SiN_x_O_y_ film, serving as a solid reference for fabrication.

The merits of using Si in integrated photonics come not only from the fabrication compatibility to CMOS technology, but also from its versatility in tuning its optical parameters, which renders itself suitable for active devices. The refractive index of Si can be tuned thermally, as utilized in [3]; in this study, Klitis et al. demonstrate active group delay control in a Si Bragg grating by creating a thermal gradient along the grating length through the metal heaters. By varying the distance between the metal heaters and the waveguides, the thermal gradient profile can be adjusted, which effectively changes the Si refractive index along the grating. Both blue and red chirps can be realized using a single design, and specific dispersion compensation can be achieved by a nanometer bandwidth filter. In addition to the thermo-optic effect, the optical constants of Si can also be varied by using the carrier injection method. Inoue et al. [7] developed a novel phase modulator based on the carrier plasma effect. The fin-type electrodes are placed at self-imaging positions of a silicon multimode interference waveguide to reduce scattering losses and relax the fabrication tolerance. The measured propagation losses and spectral bandwidth were 0.7 dB and 33 nm on a 987-µm-long phase shifter. The π-shift current of the modulator was 1.5 mA.

The active Si components serve as the building blocks for complex photonic integrations. By integrating phase shifter and multimode interferometers (MMI), a silicon-on-insulator (SOI)-based polarization controller is proposed and experimentally demonstrated in [9]. Geometrical analysis based on phasors and a Poincare sphere shows that the component can be configured as either a polarization compensator or a polarization controller. Active MZIs and micro-ring filters are also used in the work [10]. Huang et al. experimentally presented a 100 Gb/s silicon photonic WDM transmitter which consists of a passive bidirectional grating coupler and active Si components. The bidirectional grating coupler works as a beam splitter, and the split light is connected to two arms of an MZI for modulation. The modulated light is coupled to the bus waveguide through a micro-ring resonator. Four channels around 1550 nm with a channel spacing of 2.4 nm are demonstrated at 25Gb/s each channel. An even larger-scale integration of Si phase shifters can build photonic processors. In [12], researchers from Shanghai Jiao Tong University proposed a two-dimensional self-coupled optical waveguide (SCOW) mesh photonic processor to work as a rectangular unitary multiport interferometer. This photonic processor can accomplish arbitrary optical unitary transformation that has wide applications in quantum signal processing and photonic machine learning.

Although taking the spotlight of making modulators, Si is not an appropriate material as a light source due to its indirect bandgap. However, efforts to make Si luminous have never stopped. Yamada et al. [6] reported a Si-based quantum dot light-emitting diode with a peak wavelength at 620 nm. The Si-based quantum dots have the potential to replace cadmium-based quantum dots which are toxic. The quantum dots are sandwiched in multilayer structures, emitting pale-orange color with 0.03% external quantum efficiency. Moving to lower frequencies, such as the terahertz regime, an integrated THz impulse source can be realized by coupling a Si optical waveguide to a germanium-based photoconductive antenna [5]. The phosphorus-doped Ge thin film has a faster transient response speed due to the carrier lifetime reduction and antenna gap narrowing. The device has the advantage of low-cost fabrication and compact integration with on-chip excitation at the laser wavelength of 1550 nm. 

In the above approach, Si itself acts as a power delivery medium rather than an active material. The functionality of generating THz wave is achieved by heterogeneous integration with Ge. Heterogeneous integration with other materials equips Si photonics with more capabilities which cannot be realized by Si intrinsically. Another example of heterogeneous integration is a photonic memristive switch [4], implemented by the integration of Si waveguides with a phase-change material Ge_2_Sb_2_Te_5_ (GST) segment. The GST material exhibits distinct optical refractive indices and extinction coefficients in amorphous and crystalline phases. The “self-holding” capability renders the material suitable for low-energy applications, as it does not require continuous power to keep the phases. 

Beyond heterogeneous integration, Si-based compounds such as silicon nitride (Si_3_N_4_) also play an important role in integrated photonics. Compared to Si, Si_3_N_4_ does not suffer from two-photon absorption and carrier absorption, rendering itself suitable for nonlinear applications. Si_3_N_4_ used in frequency comb generation is mentioned in the review article [11]. The review of the frequency comb source mainly focuses on using the semiconductor mode-lock laser as the heart of the system, pumping the nonlinear integrated waveguides for supercontinuum generation. Carrier–envelope offset detection, stabilization, requirements for lasers, and material nonlinearity are covered in this review, followed by a future outlook on heterogeneous integration of semiconductor mode-lock lasers to achieve fully on-chip stabilized frequency combs in the near-IR region. Besides nonlinear optics, using Si_3_N_4_ as the waveguide material also gives one a greater degree of freedom in a Si platform. As shown in the work done by Sharma et al. [8], Si is used as a MEMS platform for highly efficient planar optical switching, whereas light is conducted in silicon nitride waveguides. Inverted tapers were introduced to increase the butt-coupling efficiency of the Si_3_N_4_ waveguides. Different MEMS designs were simulated and optimized. The optimum design was fabricated by commercial services and tested.

While it is impossible to cover all the research areas of silicon photonics, this Special Issue provides a humble selection of related topics with state-of-the-art results, hoping to demonstrate the recent achievements in multiple aspects. We would like to take this opportunity to thank all the contributing authors for their excellent work presented in this Special Issue. Our appreciation also goes to all the reviewing experts who dedicated their time to provide valuable comments and helped improve the quality of the submitted papers. The unconditional and generous support from the editorial staff of *Micromachines* is also highly appreciated.

## References

[1] Song J., Huang R., Zhang Y., Lin Z., Zhang W., Li H., Song C., Guo Y., Lin Z. (2019). Effect of Nitrogen Doping on the Photoluminescence of Amorphous Silicon Oxycarbide Films. Micromachines.

[2] Shi Y., He L., Guang F., Li L., Xin Z., Liu R. (2019). A Review: Preparation, Performance, and Applications of Silicon Oxynitride Film. Micromachines.

[3] Klitis C., Sorel M., Strain M.J. (2019). Active On-Chip Dispersion Control Using a Tunable Silicon Bragg Grating. Micromachines.

[4] Wang N., Zhang H., Zhou L., Lu L., Chen J., Rahman B.M.A. (2019). Design of Ultra-Compact Optical Memristive Switches with GST as the Active Material. Micromachines.

[5] Chen P., Hosseini M., Babakhani A. (2019). An Integrated Germanium-Based THz Impulse Radiator with an Optical Waveguide Coupled Photoconductive Switch in Silicon. Micromachines.

[6] Yamada H., Shirahata N. (2019). Silicon Quantum Dot Light Emitting Diode at 620 nm. Micromachines.

[7] Inoue D., Ichikawa T., Kawasaki A., Yamashita T. (2019). Silicon Optical Modulator Using a Low-Loss Phase Shifter Based on a Multimode Interference Waveguide. Micromachines.

[8] Sharma S., Kohli N., Brière J., Ménard M., Nabki F. (2019). Translational MEMS Platform for Planar Optical Switching Fabrics. Micromachines.

[9] Preite M.V., Sorianello V., de Angelis G., Romagnoli M., Velha P. (2019). Geometrical Representation of a Polarisation Management Component on a SOI Platform. Micromachines.

[10] Huang B., Zhang Z., Zhang Z., Cheng C., Zhang H., Zhang H., Chen H. (2019). 100 Gb/s Silicon Photonic WDM Transmitter with Misalignment-Tolerant Surface-Normal Optical Interfaces. Micromachines.

[11] Malinowski M., Bustos-Ramirez R., Tremblay J.-E., Camacho-Gonzalez G.F., Wu M.C., Delfyett P.J., Fathpour S. (2019). Towards On-Chip Self-Referenced Frequency-Comb Sources Based on Semiconductor Mode-Locked Lasers. Micromachines.

[12] Lu L., Zhou L., Chen J. (2019). Programmable SCOW Mesh Silicon Photonic Processor for Linear Unitary Operator. Micromachines.

